# Strategies to Reduce the Rate of Plate Waste in Hospitalized Patients: A Scoping Review

**DOI:** 10.3390/nu15020301

**Published:** 2023-01-06

**Authors:** Sangeetha Manimaran, Nurul Huda Razalli, Zahara Abdul Manaf, Arimi Fitri Mat Ludin, Suzana Shahar

**Affiliations:** 1 Nutrition Science Program, Centre for Healthy Aging & Wellness (H-CARE), Faculty of Health Sciences, Universiti Kebangsaan Malaysia, Jalan Raja Muda Abdul Aziz, Kuala Lumpur 50300, Malaysia; 2 Dietetic Program, Centre for Healthy Aging & Wellness (H-CARE), Faculty of Health Sciences, Universiti Kebangsaan Malaysia, Jalan Raja Muda Abdul Aziz, Kuala Lumpur 50300, Malaysia; 3 Biomedical Science Program, Centre for Healthy Aging & Wellness (H-CARE), Faculty of Health Sciences, Universiti Kebangsaan Malaysia, Jalan Raja Muda Abdul Aziz, Kuala Lumpur 50300, Malaysia

**Keywords:** hospital food waste, hospital plate waste, malnutrition, foodservice

## Abstract

There is evidence that hospital waste is indisputably high, and various strategies have been used to reduce the hospital’s rate of plate waste. This study aimed to map the currently implemented strategies in lowering the rate of plate waste in hospitals and categorize the different types of strategies used as interventions, as well as determine their impact based on specific parameters. The scoping review method included a search of three databases using the Preferred Reporting Items for Systematic Reviews and Meta-Analyses (PRISMA-SCR). The duplicate articles (*n* = 80) were removed. A total of 441 articles remained for the title and abstract screening. After 400 were excluded, 41 articles were reviewed for eligibility. Thirty-two full articles were eliminated due to a lack of focus on plate waste evaluation. Finally, nine accepted studies were grouped into five categories: menu modification, room service implementation, menu presentation, meal-serving system, and dietary monitoring tool. In conclusion, results showed that the majority of the studies implemented either of the five strategies to reduce plate waste; however, the cook-freeze system and staff training for both kitchen and ward staff were not yet part of any intervention strategy. The potential of this method should be explored in future interventions.

## 1. Introduction

According to the United Nations Sustainable Development Goals (SDGs), food waste is one of society’s most critical challenges [1]. A higher proportion of food waste that is generated impacts the environment, society, and economy [2]. A lesser-known fact is that the foodservice sector accounted for 14% of food waste in most affluent nations in 2010 [3], which translates to one meal wasted out of every six [4]. In the industrialized nation of the United States, about 188 kg of food waste per capita per year, with an estimated worth of $165.6 billion, is produced at the final consumption level [5]. In addition, research conducted by SWCorp in Malaysia reported that the entire quantity of food waste will be sufficient to fill sixteen Twin Towers of Malaysia by 2020 [6]. The costs of disposing of food waste include the price of purchasing raw food materials, food storage, food transportation, food preparation, labor costs, and food waste disposal costs [7].

Every foodservice organization strives to produce, deliver, and serve safe foods [8]. Hospital is one of the foodservice establishments that provide food to its patients [9]. In fact, in some healthcare facilities in New Zealand, food accounts for up to 50% of all waste. [10]. A recent review by Alshqaqeeq and colleagues [11] found that the rate of food waste in hospitals produced globally ranged from 6% to 65%. On the other hand, a previous Malaysian study indicated that depending on the food group and sex, public food waste ranged from 20% to 90% [12]. For instance, food waste tended to be highest for protein dishes such as chicken and fish and more prevalent among women for most food items [12]. Another study by Aminuddin and colleagues [13] involving government hospitals in East Malaysia reported that the average plate waste was 36% in 2017. Additionally, in a recent report, the average percentage of food waste for therapeutic diets served in 21 public hospitals was reported to be 47.4% in 2016 [14]. Recently, Razalli and colleagues [8] reported a high percentage of plate waste of 47.5% among hospitalized patients receiving a textured modified diet. The most wasted diet was found to be a blended diet (65%), followed by a minced diet (56%) and mixed porridge (35%) [8].

The amount of food waste that might be generated by the 6210 hospitals in the United States is alarming [15]. According to research by Alshqaqeeq and colleagues [16], a hospital serving 6640 patient meals per week can create more than 48,000 lbs. (24 tons) of food waste annually, including food lost during preparation and food that is prepared and not consumed or rejected by patients. In Indonesia, when documenting patients’ food intake, the majority of allied health professionals reported that plate waste was evaluated by using visual estimation from the entire tray using a Visual Comstock form, while those from smaller hospitals used a simple table in a logbook to record the proportion of food consumption [17].

Furthermore, a study conducted by Kontogianni and colleagues [18] found that about 58% of hospital patients could not consume all the foods provided to them. Based on these findings, a few factors related to food intake during hospitalization were identified, including patients’ clinical condition and food quality [18]. Physical attributes such as difficulty swallowing and food consumption are factors associated with the patient’s condition [18]. Thus, these factors have been strongly associated with increased food waste in hospitals [14]. Intestinal obstruction, dysphagia, diarrhea, nausea, vomiting, fatigue, poor appetite, being too ill or lethargic to consume food, and poor dentition are additional nutrition-impact symptoms [19]. Inadequate nutrition intake while in the hospital strongly correlated with other variables, including mealtime interruptions, avoiding meals when a meal is missed, and refusing to consume requested food [19]. As a result, every hospital foodservice must have a robust management system in place to optimize the patient’s food and nutrition intake, increase patients’ satisfaction with the foodservice and produce better quality outcomes while lowering costs and revenue generation [8]. As yet, little is known of the most optimum strategies to reduce food waste among hospitalized patients.

Therefore, the present scoping review aimed to map the currently implemented strategies in lowering the rate of plate waste in hospitals and categorize the different types of strategies used as interventions, as well as determine the impact of the strategies on other parameters, in addition to plate waste. The feasibility of the strategies applicable to low-middle-income countries (LMIC), such as Malaysia, is also discussed.

## 2. Materials and Methods

This review was conducted following the Preferred Reporting Items for Systematic Reviews and Meta-Analyses Extension for the Scoping Reviews (PRISMA-ScR) [20] and using Arksey and O’Malley’s (2005) framework [21]. The methodology was strengthened by implementing the recommendations made by Levac et al. (2010) [22] and the Joanna Briggs Institute [23] methodology.

### 2.1. Stage 1: Identifying the Research Questions

This review aimed to answer the following questions:

1. What current strategies have been implemented to reduce the rate of plate waste in hospitals?
2. What are the categories of strategies to reduce the rate of plate waste in hospitals?
3. What are the impacts of the strategies on other parameters in addition to plate waste?

### 2.2. Stage 2: Identifying Relevant Studies

#### 2.2.1. Search Terms

The search terms in this review were developed based on the PCC mnemonic framework that refers to the Population, Context, and Concept (Table 1). The search terms were then developed (Table 2). The following terminology was used in this review, including plate waste and food waste. Plate waste in hospitals refers to the amount or proportion of the food served that remains uneaten by patients [24]. Conversely, food waste is a term that describes a condition that can occur anywhere and at any time in the world during the preparation, cooking, or consumption of food [11]. Boolean operators (OR, AND), including matching and abbreviated keywords, were used to combine related keywords and terms.

#### 2.2.2. Databases

This search was conducted in May 2022 and was independently discussed and verified by two reviewers. Further discussions were made with second senior authors if a consensus was not reached. Searches on the following three databases, SCOPUS, Web of Science, and PubMed, and an additional search strategy using Google Scholar, were utilized to retrieve the potentially relevant studies.

### 2.3. Stage 3: Selection of Studies

The retrieved articles were then selected based on the inclusion and exclusion criteria. The articles were only included if they were full articles published in English from January 2011 to April 2022 that discussed strategies to reduce plate waste among patients in hospital wards. However, other articles were excluded if they were conference papers, abstracts, forums, posters, books, book series, titled books, blogs, magazines, theses, review articles, qualitative research articles, guidelines, pre-printed articles, and non-peer-reviews. Furthermore, studies that did not measure plate waste in the ward were excluded.

### 2.4. Stage 4: Data Extraction

The review team developed a chart data table to assist in data extraction. The data extracted from each study were author, year, country, category of strategy, study design, patient’s characteristics (sample size, age group, type of participants, gender), methodology (pre-intervention and strategy), outcome parameters, and findings.

### 2.5. Stage 5: Collating, Summarizing, and Reporting the Results

The extracted data were mapped and organized according to the review questions. Two independent reviewers charted data from each included study into a pre-designed data-charting template using Microsoft Excel software. Summarized data, including author, year, country, category of strategy, study design, patient characteristics, strategy, and pre-intervention, are provided in Table 2. Additionally, for the findings section, studies were grouped into a few parameters, such as (i) method to estimate plate waste, (ii) patient satisfaction, (iii) nutritional intake, (iv) meal quality, (v) food cost, (vi) portion size, and (vii) nutrition assistant role and satisfaction are provided in Table 3.

## 3. Results

### 3.1. Selection of Sources of Evidence

The study selection process was in accordance with the Preferred Reporting Items for Systematic Reviews and Meta-Analyses Extension for Scoping Review (PRISMA-SCR) [20], with full-text articles being thoroughly read to ensure that the articles met the inclusion criteria. A total of 521 articles were retrieved from the searched keywords and databases. The duplicate articles were removed (*n* = 80), and 441 articles remained for the title and abstract screening. After 400 articles were further excluded, 41 full-text articles were assessed for eligibility criteria.

Of these, thirty-two full articles were excluded for reasons such as the inclusion of dietary assessment of patients without measuring the plate waste assessment (*n* = 3), menu presentation assessment tool that did not focus on plate waste assessment (*n* = 1), linked hospitals’ foodservices to local community gardens to implement robust composting programs without focus on ward plate waste of the patients (*n* = 1), staff perspective on foodservice provision (*n* = 7), foodservice system that did not focus on plate waste assessment (*n* = 2), menu modification without plate waste assessment (*n* = 2), patient satisfaction on foodservice system (*n* = 5), an innovative cooking method that did not measure plate waste (*n* = 1), room service model without plate waste assessment (*n* = 7), the use of food audit tool that did not involve in hospital wards and examine patients’ nutritional status (*n* = 2), and hiring of foodservice dietitians without the focus on plate waste assessment (*n* = 1).

**Figure 1:**
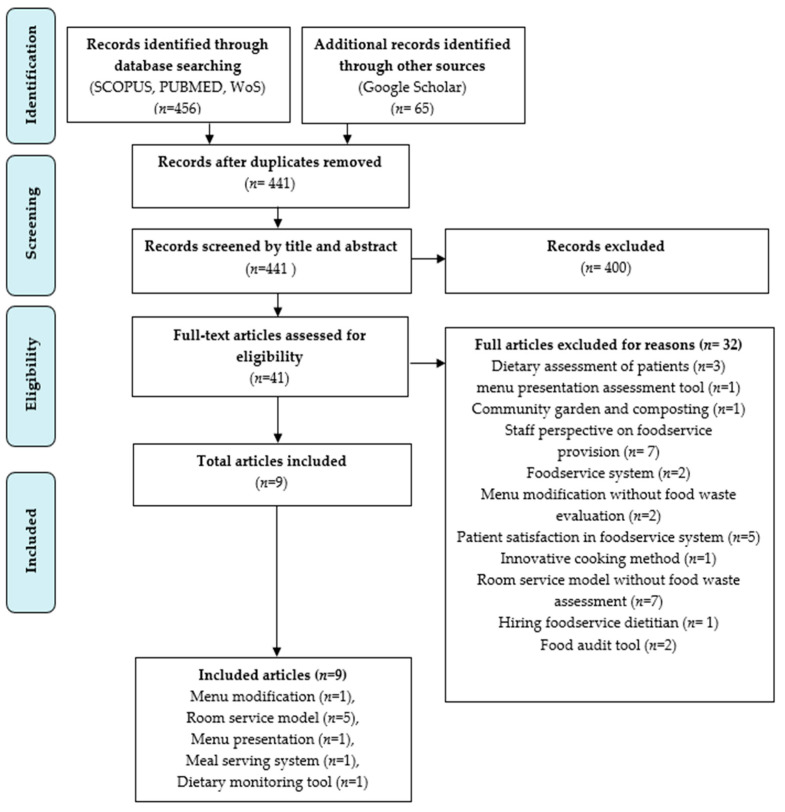
The original preferred reporting items for systematic reviews and meta-analyses for the scoping review process (PRISMA-ScR) flow diagram [20].

A total of nine articles were finally included in the review. Five categories of strategies were identified, such as (1) menu modification (*n* = 1), (2) room service model (*n* = 5), (3) menu presentation (*n* = 1), (4) meal-serving system (*n* = 1), and (5) dietary monitoring tool (*n* = 1). Based on the guidelines for conducting a scoping review, the researcher did not assess methodological quality or risk of bias in the accompanying articles [23]. Quality assessment is usually not required in scoping reviews [22,25], and this requirement is still debatable [26,27]. The PRISMA-SCR checklist is attached in Figure 1 below.

### 3.2. Study Characteristics and Patient Characteristics

**Figure 2:**
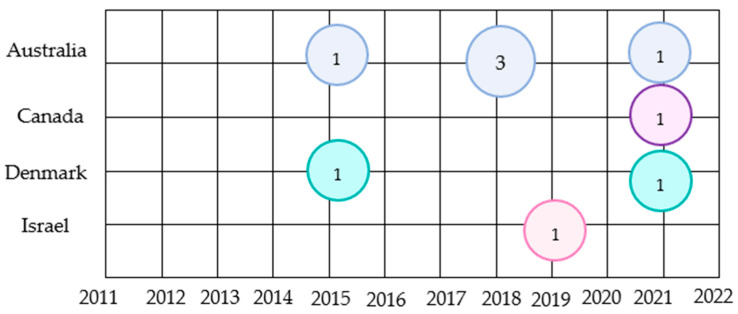
Shows the original publication years and countries of included articles (*n* = 9). Different colors of circles represent the countries. The circles correspond to the number of articles published in the year and the country.

Out of the nine included studies, eight were conducted in western high-income countries, with the majority in Australia (*n* = 5), Canada (*n* = 1), Denmark (*n* = 2), and Israel (*n* = 1). Figure 2 illustrates the publication years and countries of the included articles. The study consisted of two observational point prevalence cohort studies, a quasi-experimental study, a randomized intervention study, a pilot study, a prospective cross-sectional study, two retrospective analysis studies, and a prospective observational cohort study (*n* = 1). Two of these selected studies specifically involved geriatric patients [28,29]. The sample size of most studies ranged from a total of 65 to 200 participants.

### 3.3. Types of Strategies to Reduce the Rate of Plate Waste in Hospitals

This review included five strategies implemented as interventions to reduce the rate of plate waste in hospitals. The strategies included menu modification, room service model, menu presentation, meal-serving system, and dietary monitoring tool. A summary of the interventions and pre-intervention is provided in Table 2. A total of five studies were found on room service model strategy to combat plate waste problems in hospitals, including two observational point prevalence cohort studies [30,31], two retrospective analyses [32,33], and one quasi-experimental study [34] that examined a comparison between a variety of room service models and the bought-in, thaw-retherm foodservice model and the traditional and cook-fresh paper menu system in hospitals. The investigated strategies included an à-la-carte-style menu with varied items and ordering times that suited the patients [32,33] and a 24 h available patient-directed bedside electronic meal ordering system using a touch screen and/or mouse and keyboard [30]. Furthermore, a bedside meal ordering system model was identified that allowed nutrition assistants to discuss suitable meal choices according to patients’ preferences and enter orders in a handheld wireless mobile device (Apple iPad) with a 7-day cycle menu of freshly cooked contemporary menu items [31]. Furthermore, Dining on Call (DOC), also known as room-service-style dining, enables patients to conveniently order meals anytime from a single integrated menu and can be delivered within 45 min [34].

Recently, a study focusing on menu modification strategy in Denmark [29] found that Free Choice Menu (FCM) allowed patients to order from a menu that included both hot and cold foods 24 h a day and improved cooking methods. Another study that highlighted a meal presentation intervention strategy used an orange napkin to improve food presentation and dietary intake and reduce readmission rates of hospital patients [35]. Furthermore, the Dietary Intake Monitoring System (DIMS) strategy is an innovative tool to monitor plate waste for portion size of meals and reduce food waste. The technology captures photographs while simultaneously collecting data on the dish’s weight, food temperature, date, and time [28]. Finally, in another study on the meal-serving system, the oral intake of adults in an acute care institution was improved by using smooth, pureed foods that were thickened and molded into a three-dimensional form [36].

**Table 2:** Summary of strategies implemented as pre-interventions and interventions to reduce the rate of plate waste in hospitals and the characteristics of patients.

Author/Year/Country	Category of Strategy	Study Design	Patient’s Characteristics	Strategy	Pre-Intervention
Abou et al., 2021 (Canada) [34]	Room service model	Quasi-experimental study	The pre-DOC surveyed a total of 65 children and 68 adult female patients, while for the post-DOC survey, a total of 70 children and 70 adult female patients from BC Children’s and Women’s Hospitals completed the survey At NYGH, 33 patients participated in the pre-post Dining on Call (DOC) survey	**Dining on Call (DOC) hospital foodservice** Patients can request meals at any time of day Meals delivered within 45 min	**Traditional foodservice** Limited choice of menus Food trays are delivered at specific times
Barrington et al., 2018 (Australia) [30]	Room service model	An observational point prevalence cohort study	**For paper menu** Involved a total of 96 oncology patients 54% males with a mean age of 60.5 (19–93) **The bedside meal ordering system (BMOS)** Involved a total of 105 oncology patients 53% males with a mean age of 65.0 (18–88)	**Patient-Directed Bedside Electronic Meal Ordering System (BMOS)** Computerized terminals were available 24 h Patients choose meals at any time of the day Order up to 1 h before a meal	**Paper Menu (PM)** Ordered meals the day before A default meal (no choice) would be served if the patient did not complete or update their order No nutritional information or food allergies were included except made based on dietetic recommendation
Dynesen et al., 2021 (Denmark) [29]	Menu modification	A prospective cross-sectional study	**Traditional trolley meal service** 98 geriatric ward patients 57% females and 43% males Mean age of 84.8 (6.8) **Free Choice Menu (FCM)** 52 geriatric ward patients 58% females and 42% males Mean age of 83.4 (7.5)	**Free Choice Menu (FCM)**. Food was reheated in microwave ovens before serving Variety choice of menu Orders 24 h a day Fixed meal portion sizes for energy and protein	**Traditional trolley meal service (trolley)** Limited choices of foods Orders at fixed hours Meals were served within 1–3 h of cooking
Farrer et al., 2015 (Australia) [36]	Meal-serving system	A pilot study	**Molded meals (treatment group)** 27 adult patients in an acute care institution 37% males and 63% females Aged below 65 years 22% and above 65 years old 78% **Non-molded meals (control group)** 38 adult patients in an acute care institution 79% males and 21% females Aged below 65 years (24%) and above 65 years old (76%)	**Molded smooth pureed meals** Thickened and molded to a three-dimensional form	**Non-molded smooth pureed meals** Served in the standard format
Navarro et al., 2019 (Israel) [35]	Meal presentation	A randomized intervention study	**White napkin (control group)** 65 adult patients from the internal medicine department 55.4% females and 44.6% males Mean age of 79 years old (19) **Orange napkin (experimental group)** 66 adult patients from the internal medicine department 9.4% females and 60.6% males Mean age of 68 years old (28)	**The addition of an orange napkin (experimental group)** Cost approximately USD 0.05 for each napkin for the hospital meal tray	**White napkin (control group)** Received usual food trays with a white napkin
Neaves et al., 2021 (Australia) [32]	Room service model	A retrospective analysis	**Thaw retherm service model** 134 adult patients from surgical, thoracic, and cystic fibrosis wards 47% females and 53% males The median age of 58.7 years old (29) **On-demand room service model** 76 adult patients from surgical, thoracic, and cystic fibrosis wards. 48.7% females and 50.3% males and 1.3% unspecified gender The median age of 73.6 years old (28)	**On-demand room service model** An à la carte restaurant-style menu Order meals through a call center at a time that suits patients between 6.30 a.m. and 7.00 p.m. Meal delivered within 45 min of the order	**Bought-in, thaw-retherm foodservice model and cook-fresh** Menu items purchased frozen in bulk were used in the thaw-retherm Freshly prepared items such as salads and sandwiches, a limited selection of hot breakfast meals designed specifically for high-protein diets
McCray et al., 2018a (Australia) [33]	Roomservice model	A retrospective analysis	**Traditional foodservice model** 84 adult patients from the general medical, surgical, and cancer wards 57% females and 43% males Mean age of 63.4 years old (19.1) **Room service model** 103 adult patients from the general medical, surgical, and cancer wards 52% females and 48% males Mean age of 70.4 years old (15.0)	**Room service (RS)** Patients order through room service representatives in a central call center Meal orders between 06.30 a.m. to 07.00 p.m. A single integrated à la carte menu Meal delivered within 45 min	**Traditional Model (TM)** Patients ordered meals by filling out a paper menu Cook fresh, 14-day cycle menus Breakfast is served between 06.30 a.m. to 07.30 a.m., lunch between 11.45 a.m. to 12.45 p.m., and supper between 17.00 p.m. to 18.00 p.m.
McCray et al., 2018b (Australia) [31]	Room service model	An observational point prevalence	**Traditional paper menu ordering system (TM)** 84 adult patients from the general medical, surgical, and oncology wards 57% females and 43% males Mean age of 63 ± 19 **Bedside meal ordering system (BMOS)** 104 adult patients from the general medical, surgical, and oncology wards 56% females and 44% males Mean age of 72 ± 15.	**Bedside meal ordering system (BMOS) model** The nutrition assistant will discuss the meal choice according to the patient’s preferences and enters orders into a handheld wireless mobile device (Apple iPad) A 7-day cycle menu of cooked fresh and more contemporary menu items is provided	**Traditional paper menu system (TM)** The nutrition assistant delivers and collects paper menus from patients for dinner the same day, whereas breakfast and lunch the following day. Manually processes the information and delivers it to the kitchen for production A 14-day cycle cook-fresh menu.
Ofei et al., 2015 (Denmark) [28]	Dietary intake monitoring tool	A prospective observational cohort study	**A trolley meal delivery system with dietary intake monitoring system (DIMS) technology** 71 patients from medicine, surgery, and oncology wards **Nutritional at risk** 55% females Mean age of 62.9 ± 15.2 **Nutritional not at risk** 50% females Mean age of 66.4 ± 11.1	**Utilize a trolley meal delivery system with dietary intake monitoring system (DIMS) technology** Monitor portion sizes, nutritional intake, and plate waste in daily practice Captures photographs while simultaneously collects data on the weight, food temperature, date, and time.	**Trolley meal delivery system** Allows hospital patients to select food items and portion sizes directly from the food trolley

**Table 3:** The impact of the strategies on other parameters and plate waste.

Author/Year/Country	Parameter	Findings				
Abou et al., 2021 (Canada) [34]	Tray waste (visual estimation) Patient satisfaction Food cost and labor cost per meal per day	**Tray waste**		**Patient Satisfaction**		**Food cost and labor cost per meal per day**
		Tray waste was reduced from 18% pre-DOC to 8.9% post-DOC implementation for BC Children’s and Women’s hospitals For NYGH, there was a reduction in tray waste of 24% for breakfast, 5% for lunch, and 10% for dinner post-DOC		At BC Children’s and Women’s hospitals, post-DOC results showed an increase of 1.9% and 7.9% in overall patient satisfaction for children and women, respectively In NYGH, overall patient satisfaction increased by 7%.		At BC Children’s and Women’s Hospitals, the cost of food per meal per day was reduced by 43.5% post-DOC and the cost of labor per meal per day decreased by 33.4% post-DOC At NYGH, food and labor costs per meal per day increased by 7.8% and 0.8%, respectively
Barrington et al., 2018 (Australia) [30]	Plate waste (visual scale) Dietary intake (visual scale) Patient meal experience	**Plate waste**		**Dietary intake**		**Patient meal experience**
		No significant difference between the average daily plate waste between the two cohorts (35.4% and 34.3%, respectively) (*p* = 0.75)		There was a significant increase in average energy intake (6457 kJ day^−1^ versus 4805 kJ day^−1^, *p* < 0.001) and protein intake (73 g day^−1^ versus 58 g day^−1^, *p* < 0.001)		60% of the patients accessed the BMOS independently The BMOS cohort had a significant increase in receiving the ordered food (*p* < 0.001) and choosing the preferred food (*p* = 0.006)
Dynesen et al., 2021 (Denmark) [29]	Plate waste (weighing method) Nutritional intake and recordings of dietary intake (3-day weighing method) Portion size	**Plate waste**		**Nutritional intake**		**Portion Size**
		There was no significant difference (*p* = 0.25) in total food waste between the two concepts A significantly lower (*p* = 0.0005) lunch plate waste for the FCM concept (15.6%) compared with the trolley concept (26.1%) There were no significant differences in plate waste between the two concepts for breakfast (*p* = 0.95) or dinner (*p* = 0.62).		The difference between the concepts were not significant for either energy intake (*p* = 0.498) or energy coverage (*p* = 0.51) The difference between the concepts were not significant for either protein intake (*p* = 0.0588) or protein coverage (*p* = 0.11)		A significant positive relationship between portion size and meal consumption percentage for both concepts (trolley: *p* < 0.000; FCM: *p* = 0.031)
Farrer et al., 2015 (Australia) [36]	Plate waste (Weighing Method) Patient satisfaction	**Plate Waste**				**Patient Satisfaction**
		There was a significance in the number of patients increasing oral intake from <1/4 meal eaten to >3/4 meal in molded form (*p* = 0.03) The median plate waste was reduced by over 100 g in the molded form (treatment group). There was no significant(*p* = 0.09) change overall. The main reasons for the food waste reported in the survey were clinical reasons comprising of 84% (*n* = 31) in the control group and 81% (*n* = 22) of the treatment group or ‘food issues’, 16% (*n* = 6) in the control group and 19% (*n* = 5) of the treatment group.				No statistical significance was seen in the hedonic rating of patient satisfaction with meals in the molded form as compared to the control group (*p* = 0.31)
McCray et al., 2018a (Australia) [33]	Plate waste (visual scale) 5-point scale Nutritional intake (visual scale) Patient satisfaction Food cost	**Plate waste**	**Patient Satisfaction**	**Nutritional intake**		**Food cost**
		Total average plate waste decreased from 30% to 17% (*p* < 0.001)	Patient satisfaction indicated an improvement in room service, with 98% of patients scoring the service as good to very good compared to 75% for the traditional model (*p* < 0.04)	The nutritional intake increased statistically significant with room service in both energy intake (5513 kJ day^−1^ versus 6379 kJ day^−1^, *p* = 0.020) and protein intake (53 g day^−1^ versus 74 g day^−1^, *p* < 0.001) The energy and protein intake as a percentage of requirements (64% versus 78%, *p* = 0.002 and 70% versus 99%, (*p* < 0.001, respectively)		Patient food costs decreased by 28% per year with room service
McCray et al., 2018b (Australia) [31]	Plate waste (5-point visual scale) Patient satisfaction Nutritional intake Nutrition assistant role and satisfaction Patient food costs	**Plate waste**	**Patient Satisfaction**	**Nutritional** **intake**	**Nutrition assistant role and satisfaction**	**Food cost**
		The mean plate waste decreased significantly from 30% to 26% (*p* < 0.001)	84% of subjects preferred the bedside meal ordering system (BMOS) (*p* = 0.00)	There was a significant increase in mean daily energy (*p* = 0.035) and protein intake (*p* < 0.001)	A total of 86% of nutrition assistants preferred the BMOS for post-implementation (*p* = 0.047)	Comparable 12-month period showed a decrease in total patient food costs by 19% for the BMOS compared to TM.
Navarro et al., 2019 (Israel) [35]	Plate waste (modified Comstock plate waste scale) Patient satisfaction	**Plate Waste**		**Patient Satisfaction**		
		Patients in the orange napkin group (*n* = 66) (experimental) consumed 17.6% more hospital-provided food than those in the white napkin (control) group (*n* = 65), (*p* = 0.002) The proportion of lunch consumed remained higher in the orange napkin group than in the control group (54.34 ± 4.08 versus 31.86 ±4.12; *p* = 0.004)		Patients in the orange napkin group also reported significantly greater satisfaction with the hospital foodservice (*p* < 0.0001) Patients in the orange napkin group had higher foodservice satisfaction scores for all aspects queried food, such as service satisfaction, favored patient’s lunch, satisfactory food, food presentation, preservation of cold meals, provision of meals (breakfast, lunch, and dinner), adequate time to consume a meal, and acceptable appearance of lunch (*p* < 0.0001)		
Neaves et al., 2021 (Australia) [32]	Plate waste (visual method-5 point visual scale) Food cost Patient satisfaction Nutritional intake (visual method) 5 consecutive weekdays Meal quality	**Plate waste**	**Patient Satisfaction**	**Nutritional intake**	**Meal quality**	**Food cost**
		Overall mean plate waste reduced from 40% to 15% Total average production waste measured decreased from 15% for thaw-retherm to 6% for room service (t [33] = 6.908, *p* < 0.001)	Patient satisfaction with room service increased from 75% to 89.8% (χ^2^ 9.985 [2]; *p* = 0.007)	Average energy and protein intake, as well as percentage requirements, improved between thaw-retherm and room service (4320 kJ/day vs. 7265 kJ/day; 42.4 g/day vs. 82.5 g/day; and 46% vs. 80.7%; 49.9% vs. 98.4%; all *p* < 0.001)	There was a significant improvement in the appearance of meals (*p* = 0.031)	Food costs decreased by 9% for room service
Ofei et al., 2015 (Denmark) [28]	Plate waste Nutritional intake	**Plate waste**		**Nutritional intake**		
		A positive relationship between meal portion size and plate waste (*p* = 0.002) and increased food waste in patients with nutritional risk during supper (*p* = 0.001)		For lunch, the percentage of energy consumed by patients at risk was 9 (4–16%), and for patients not at risk 11 (6–17%) and not statistically significant (*p* = 0.150). The percentage of protein consumed by patients at risk was 8 (3–15%), and for patients not at risk, 11 (5–19%). It was also not statistically significant (*p* = 0.09). For supper, the percentage of energy consumed by patients at risk was 10 (6–21%), and for patients not at risk, 13 (9–19%) and not statistically significant (*p* = 0.143). The percentage of protein consumed by patients at risk was 12 (5–23%), and for patients not at risk, 15 (8–22%) and not statistically significant (*p* = 0.698).		

### 3.4. The Impacts of the Strategies on Other Parameters and to Plate Waste

#### 3.4.1. Method to Estimate Plate Waste

Plate waste in hospitals refers to the volume or percentage of food served to patients that have been discarded [24]. In the hospital context, there are two methods of measuring plate waste: the weighing method and the visual estimation method. Hence, this review of nine studies focused on plate waste measurement.

The weighing method is the most precise option, but it is time-consuming and resource-intensive to implement without disrupting or delaying typical foodservice operations [24]. A total of two studies were conducted using the weighing method [29,36]. In a study that implemented the food-serving-style strategy, the patients received either a non-molded (control group) or a molded smooth pureed lunch (treatment group). Overall, the control group generated 286 g of plate waste, while the treatment group generated 160 g [36].

Next, the Free Choice Menu (FCM) strategy for menu modification evaluated that plate waste using the weighing method was significantly lower (*p* = 0.0005) for lunch (15.6%) compared to the trolley concept (26.1%) [29].

Meanwhile, a visual estimation method is used to estimate the amount of food that remained. A variety of scales have been proposed. A seven-point scale (all, one mouthful eaten, ¾, ½, ¼, one mouthful remaining, none) [37] and the Comstock 6-point scale (all, one bite taken, ¾, ½, ¼, one mouthful left, none) [38] are the most comprehensive measurements, followed by the five-point scale (all, ¾, ½, ¼ or less, none, or virtually none) [39], a four-point scale (all, ½, ¼ or fewer, none or almost none) [40] and a three-point scale (all, below 50%, above 50%) [41].

A study conducted by Navarro et al. [35] used the Modified Comstock Plate Waste 6-point scale (0%, 25%, 50%, 75%, 90%, or 100%) to determine the proportion of the menu item remaining on the plate. Patients in the group using the orange napkin (*n* = 66) consumed 17.6% more hospital-provided food than those in the white napkin (control) group (*n* = 65), (*p* = 0.002), driven by the significantly higher proportion of carbohydrate side dishes (*p* = 0.015) and vegetable dishes consumed (*p* = 0.022) [35]. The proportion of lunch consumed remained higher in the orange napkin group than in the control group (54.34 ± 4.08 versus 31.86 ±4.12; *p* = 0.004) [35].

Besides, the study conducted by Ofei et al. [28] revealed a significant relationship between the portion size of the meal and plate waste (*p* = 0.002). For the patients with nutritional risk, the food waste increased significantly during supper (*p* = 0.001) [28].

Furthermore, a total of five studies used the room service model [30,31,32,33,34], which allowed patients to make an ordered meal based on their preference at any time of the day with a variety of choices using the visual method five-point visual scale (0%, 25%, 50%, 75%, 100%) to evaluate plate waste. In four out of the five studies, the total average percentage of plate waste decreased from 40% to 5% and was found to be statistically significant (*p* < 0.001) [31,32,33,34].

#### 3.4.2. Patient Satisfaction

In previous studies conducted by [31,32,33], patient satisfaction was measured using the validated Acute Care Hospital Food Service Patient Satisfaction Questionnaire [42]. For the room service model, patient satisfaction increased from 75% to 89.8% (χ^2^ 9.985 [2]; *p* = 0.007) [32], with 98% of the patients provided a score from good to very good, compared to 75% for the traditional model (*p* < 0.04) [33]. The overall foodservice satisfaction remained constant, with significantly more patients preferring bedside menu ordering systems (84%), while (16%) of patients preferred the traditional model (*p* = 0.00) [31].

A study by Navarro and colleagues [35] observed patient satisfaction levels using the Utah State University Hospital Food Service Patient Satisfaction Survey. Patients from the orange napkin group revealed significantly higher levels of satisfaction with the hospital dining service (*p* < 0.0001). They had higher foodservice satisfaction scores for serving patient’s preferred lunch, satisfactory food, appetizing presentation, preservation of served cold food, provision of main meals (breakfast, lunch, and dinner), adequate time to consume the meal, and lunch looks tasty with (*p* < 0.0001) [35].

Regarding the perceptual ratings for pureed and molded lunches for individuals with and without impaired swallowing [36], no statistical significance was found in the hedonic rating of patients’ satisfaction with meals in the molded form compared to the control group (*p* = 0.31). A bedside satisfaction survey was verbally given to patients by a patient satisfaction surveyor and was used to compare patients’ satisfaction with pre- and post-Dining on Call (DOC). The management of hospital foodservice operations regularly uses this kind of survey [34]. At BC Children’s and Women’s hospitals, post-DOC results showed an increase of 1.9% and 7.9% in overall patient satisfaction for children and women, respectively [34]. According to patient satisfaction surveys in NYGH, overall patient satisfaction increased by 7% [34].

#### 3.4.3. Nutritional Intake

Two studies investigated the effectiveness of nutritional intake by implementing a room service model using an à la carte menu [32,33] that was compared with a paper menu (cooked fresh, 14-day cycle menu), bought-in, thaw-retherm foodservice model and cooked fresh food, respectively. Both of the studies reported that the nutritional intake of patients had increased statistically significantly in room service for both energy intake (5513 kJ day^−1^ versus 6379 kJ day^−1^, *p* = 0.020) and protein intake (53 g day^−1^ versus 74 g day^−1^, *p* < 0.001) intake [33] with average energy and protein intake, percentage requirements, improved between thaw-retherm and room service (4320 kJ/day vs. 7265 kJ/day; 42.4 g/day vs. 82.5 g/day; and 46% vs. 80.7%; 49.9% vs. 98.4%; all *p* < 0.001), respectively [32].

The 7-day cycle menu [31], Free Choice Menu (FCM) [29], and Patient-Directed Bedside Electronic Meal Ordering System (BMOS) [30] were provided with a variety of meal choices according to the clinical conditions of the patients. The systems also enabled patients to make orders at any time according to personal preferences and were compared with traditional trolley meal service [29] orders at fixed hours as well as traditional paper-based menu systems [30,31], which have to make an order a day before. The study shows there was a significant increase in mean daily energy and protein intake (*p* = 0.035; *p* < 0.001 respectively) [31], average energy intake (6457 kJ day^−1^ versus 4805 kJ day^−1^, *p* < 0.001), and protein intake (73 g day^−1^ versus 58 g day^−1^, *p* < 0.001) [30].

Another study applied the trolley meal delivery system with and without the dietary intake monitoring system (DIMS) technology. For lunch, the energy intake (*p* = 0.150) and protein intake (*p* = 0.09) of patients were not statistically significant. In addition, the energy intake (*p* = 0.143) and protein intake (*p* = 0.698) for supper were not statistically significant [28].

#### 3.4.4. Meal Quality

The meal quality scores were high for both the thaw-retherm and room-service models. There was a significant improvement in the appearance of meals (*p* = 0.031) [32].

#### 3.4.5. Food Cost

A total of four studies on the room service model strategy reported a reduction in food costs ranging from 9% to 43.5% compared to the traditional meal service system [31,32,33,34].

#### 3.4.6. Portion Size

A study conducted by Dynesen and colleagues [29] shows there was a significant positive relationship between portion size and meal consumption percentage for both concepts (trolley: *p* < 0.000; Free Choice Menu: *p* = 0.031).

#### 3.4.7. Nutrition Assistant Role and Satisfaction

During the pre-implementation of the bedside meal ordering system (BMOS), approximately 36% of nutrition assistants preferred this strategy, and this increased significantly to 86% during post-implementation (*p* = 0.047) [31].

## 4. Discussion

The purpose of the scoping review is to map the current strategies implemented to reduce the rate of food waste in hospitals, categorize the strategies used, and determine the impact of the strategies on other parameters and plate waste. A total of five categories of strategies were identified, including menu modification, room service model, menu presentation, meal-serving system, and dietary monitoring tool.

The ideal strategy that can be adopted to reduce hospital plate waste is the room service model. However, we also acknowledge the challenges of implementing this strategy, particularly in hospitals in middle-to low-income countries, implying that enforcing a cook-freeze system and staff training are worthwhile strategies to combat food waste.

The room service model involves organizing structured mealtime and meal production schedules and mainly focuses on patients’ diagnosis and treatment schedules [33]. Additionally, ordering through the call center or bedside meal order personnel can promote better patient interactions and patient participation [33]. Furthermore, healthcare personnel should be responsible for supporting patients to make the best decisions when ordering the meal [31,43]. It is also likely that allowing ordering nearer to mealtimes can better accommodate patients’ current preferences, boost satisfaction, and permit food delivery within 45 min of ordering [31,32,33,34]. However, it will be more challenging to implement this strategy in low to middle-income countries such as Malaysia as most hospitals are high-volume and public- or government-funded. Thus, the cost will be a critical issue.

The cook-freeze method will be more effective in reducing plate waste in public or government-funded hospitals where the conventional foodservice system is common. Cook-freeze is similar to the cook-chill method, except that the prepared meals are frozen immediately rather than frozen in a blast freezer [44]. To increase menu flexibility, items can be frozen in bulk or as individual servings, especially for individuals who have specific nutritional requirements, such as gluten-free [44]. In a trolley, hot food may also be delivered to wards so that patients can make their selections there. The aroma and presentation of the food may help with patients’ hunger, whereas various serving sizes and food choices based on current appetite can be offered [44]. Additional nursing staff can be involved in notifying patients of the arrival of the trolley, further encouraging and socializing patients and more. However, disadvantaged patients may not have the mobility to access the trolley; thus, managing therapeutic diets can indeed be challenging because foodservice staff are untrained in this area, leading to more food waste (from the bulk trolley but not from individual patient meal plates) due to the number of options that must be included in the trolley to cover the menu [45]. In addition, we also identified that staff training had not been reported as a strategy to combat plate waste problems. It is a plausible strategy worth exploring since staff training has been linked with better service quality [46]. Understanding hospital foodservice management is one of the strategies to enhance hospital food provision [47]. A few studies focused on strategies such as the inclusion of room service [30,31,32,33,34], menu modifications [29], meal-serving style [36], menu presentation [35], and the dietary intake monitoring tool [28], but none of the studies intervened in staff training.

Consequently, two studies that were conducted in Malaysia [47,48] emphasized hospital personnel’s role in food provision and investigate staff attitudes and behaviors during patients’ mealtimes. The viewpoints and experiences of important stakeholders aided in the development of knowledge in several aspects of hospital meal production that affect patients’ decisions to accept and eat food.

There are a few strengths and limitations in this review. The review was conducted twice to filter duplicate articles during extraction and consider the study eligibility to meet specific criteria. Furthermore, this is the first review focused on documenting strategies to reduce plate waste in hospitals. This limitation of the study was the absence of a quality assessment, which is not a requirement for scoping reviews; therefore, it was not included in the methodology. In addition, very few publications focused on strategies to combat plate waste problems in hospitals, and the majority of studies were conducted in western high-income countries such as Australia, Canada, Denmark, and Israel. Therefore, more studies on strategies to reduce plate waste in hospitals in low to middle-income countries are needed to link with sustainable development goals related to food sustainability and the environment.

## 5. Conclusions

An appropriate solution proven to contribute to the objective of this study is to reduce food waste through plate waste, which has been reported to be the largest rate in a hospital setting [24]. It has been postulated that some food waste is inevitable in order to satisfy the dietary and nutritional needs of patients. [44]. The distribution of food to patients and the accompanying levels of waste are frequently prioritized in cost-cutting efforts. Food waste can be caused by a variety of variables, including food-service model design such as bulk cooking and reheating, extended lead time forecasting, and in-advance meal ordering, missed meals related to environmental factors, specifically hospital procedures and test schedules, as well as individual patient problems in the case of reduced appetite and other impacts of clinical symptoms and treatments, such as nausea or pain [44].

This review has identified five strategies that can potentially reduce the rate of plate waste in hospitals, such as menu modification, room service model, menu presentation, meal-serving system, and dietary monitoring tool. The room service model is the most suitable strategy to combat food waste issues in conventional foodservice systems. However, it would be challenging to implement and apply it in government-funded hospitals in low- and middle-income countries (LMIC), such as Malaysia, as the cost will be the major challenge. In conclusion, the cook-freeze system and staff training are not yet part of any intervention strategy to combat the plate waste problem. Training healthcare staff could be explored in the near future and should include a discussion on the obligations and duties required in foodservice operations. It is recommended that the government regularly examine foodservice staff competency, budget food allocations, staff training, and replace equipment used in kitchens to improve the overall foodservice system and cost-effectiveness in reducing the rate of plate waste in hospitals.

## Figures and Tables

**Table 1:** Keywords used for this review.

	Searched Terms
Concept	strategy OR policy OR guidelines OR procedure AND food service OR meal service AND
Context	hospital food waste AND hospital plate waste
Population	AND hospitalized patients

## Data Availability

Not applicable.
